# Analysis of Consistency of Transthoracic Bioimpedance Measurements Acquired with Dry Carbon Black PDMS Electrodes, Adhesive Electrodes, and Wet Textile Electrodes

**DOI:** 10.3390/s18061719

**Published:** 2018-05-26

**Authors:** Hugo F. Posada-Quintero, Natasa Reljin, Caitlin Eaton-Robb, Yeonsik Noh, Jarno Riistama, Ki H. Chon

**Affiliations:** 1 Department of Biomedical Engineering, University of Connecticut, Storrs, CT 06269 USA; natasa.reljin@gmail.com (N.R.); caitlin.eaton_robb@uconn.edu (C.E.-R.); ki.chon@uconn.edu (K.H.C.); 2 College of Nursing, University of Massachusetts Amherst, Amherst, MA 01003, USA; ynoh@umass.edu; 3 Department of Electrical and Computer Engineering, University of Massachusetts Amherst, Amherst, MA 01003, USA; 4 Philips Research, 5656 Eindhoven, The Netherlands; jarno.riistama@philips.com

**Keywords:** bioimpedance, electrocardiogram, heart failure, electrodes, fluid accumulation

## Abstract

The detection of intrathoracic volume retention could be crucial to the early detection of decompensated heart failure (HF). Transthoracic Bioimpedance (TBI) measurement is an indirect, promising approach to assessing intrathoracic fluid volume. Gel-based adhesive electrodes can produce skin irritation, as the patient needs to place them daily in the same spots. Textile electrodes can reduce skin irritation; however, they inconveniently require wetting before each use and provide poor adherence to the skin. Previously, we developed waterproof reusable dry carbon black polydimethylsiloxane (CB/PDMS) electrodes that exhibited a good response to motion artifacts. We examined whether these CB/PDMS electrodes were suitable sensing components to be embedded into a monitoring vest for measuring TBI and the electrocardiogram (ECG). We recruited *N* = 20 subjects to collect TBI and ECG data. The TBI parameters were different between the various types of electrodes. Inter-subject variability for copper-mesh CB/PDMS electrodes and Ag/AgCl electrodes was lower compared to textile electrodes, and the intra-subject variability was similar between the copper-mesh CB/PDMS and Ag/AgCl. We concluded that the copper mesh CB/PDMS (CM/CB/PDMS) electrodes are a suitable alternative for textile electrodes for TBI measurements, but with the benefit of better skin adherence and without the requirement of wetting the electrodes, which can often be forgotten by the stressed HF subjects.

## 1. Introduction

Transthoracic Bioimpedance (TBI) measures fluid accumulation in the lungs, and it is an indirect approach to detecting intrathoracic volume retention [1,2]. The measurement of TBI could be useful for patients with heart failure (HF), in whom exacerbations can manifest as a fluid build-up in the thorax. Four electrodes are commonly used for a bioimpedance measurement. Two electrodes are used for injecting the alternating current (amplitude typically in the range 100 µA to 10 mA, frequency ranging from 10 kHz to 1 MHz for multiple-frequency devices, and around 50–70 kHz for single-frequency devices), and the other two are used for measuring the resulting voltage drop. By knowing the injected current and by measuring the voltage across the electrodes, the body impedance can be calculated using Ohm’s law.

The multiple benefits of using four electrodes instead of two have been documented [3,4,5], but the primary reason is that the contact impedance between the skin and the electrodes will be cancelled in the measured responses, assuming that these are similar in all four of the electrodes [3]. This increases the sensitivity of the measurement apparatus, since the hand-to-hand bio-impedance is typically over 500 Ohms, in the thorax the bio-impedance is a decade lower, and the contact impedance of the electrodes can be several kilo-Ohms [3].

Several TBI measurement devices claim to be able to measure fluid accumulation from the skin, using adhesive electrodes; however, the repeatability of electrode placement on the skin is often poor, and the misalignment of the electrode positions leads to highly variable results. Requiring a skilled professional or another person to help to place the electrodes for anatomical consistency increases the cost, reduces usability, and makes the system less suitable for daily home use by heart failure (HF) patients. To overcome this limitation, some studies have proposed using textile electrodes that are embedded into a wearable vest [6,7,8]. Wetting the textile electrodes improves the signal fidelity, because their impedance is several orders of magnitude higher than that of the hydrogel Ag/AgCl electrodes [9]. However, requiring older and ailing patients to remember to wet the electrodes with each use might not be realistic, is inconvenient, and it is often uncomfortable for them. They may also forget to wet one or more of the electrodes, resulting in a significant impedance-imbalance. Thus, the wetting requirement is a major limitation of the textile-based electrode bioimpedance monitors. Moreover, the textile electrodes might shift with body movements, introducing further variability in the impedance measurement outcomes [7,8]. 

Recently, we developed reusable dry carbon black electrodes that did not require wetting prior to collecting the bioelectric signals, had good adhesion to the skin without adhesives, and exhibited a good tolerance to the motion artifacts [10,11]. We proposed the use of dry carbon black electrodes that were incorporated into an HF monitoring vest for daily monitoring of TBI, vital signs (e.g., heart rate), and atrial fibrillation (AF). This vest could overcome the current problems of the non-repeatability of TBI measurements by offering an easy and repeatable daily electrode placement on the skin, thanks to the vest structure; less electrode slippage on the skin, thanks to the good adhesion of the carbon black electrode material; and larger contact surface area of the electrode to the skin than the traditional adhesive electrodes. More importantly, most of the subjects with HF are very ill and often suffer from cognitive impairment, hence, they may not have the mental fortitude to remember to wet the textile electrodes prior to daily use. 

The incidence of AF and HF are alarmingly increasing in the U.S., despite advances in the treatment of cardiovascular disease [12,13]. HF is associated with substantial morbidity, mortality, and an impaired quality of life [14]. AF is the most common arrhythmia that is encountered in clinical practice and affects 3–6 million Americans [15]. HF and AF frequently coexist [15]. Patients with HF who develop AF are at a higher risk for HF, hospitalization, stroke, and death [16,17]. 

Since the symptoms of HF are dominated by shortness of breath from pulmonary edema, HF detection requires the measurement of lung fluid [17,18]. The current home health HF surveillance programs in ambulatory HF patients rely on telehealth technologies to monitor selected symptoms [19,20]. Most of these systems use daily patient questionnaires to track the symptoms, with measurement devices for monitoring physiological variables, including body weight, blood pressure, and potentially single-lead ECG and oxygen saturation. Given that only a minority of the patients with HF have significant hemodynamic perturbations, the predictive accuracy of these technologies is marginal [17,21]. Furthermore, the lead time for detecting decompensation is frequently shorter than what is clinically optimal for these systems.

The most widely-used method for HF exacerbation detection includes the use of a scale to detect fluid accumulation. Although it is non-invasive and inexpensive, this approach is not ideal because weight change is also influenced by factors other than fluid accumulation; weight change correlates weakly with HF symptomatology, which is a major driver of rehospitalization, and weight change often occurs only 2–3 days before the maximum symptomatology [22,23]. This warning window is often insufficient to prevent HF hospitalization, because telehealth providers may need an even earlier detection of fluid accumulation in order to obtain prescriptions for appropriate therapies and act on them. Efforts that are focused on the use of weight change for the early diagnosis of HF exacerbation and for preventing rehospitalization have shown mixed results, are expensive, and are labor-intensive, because they often require almost daily contact between health care professionals and patients. Despite these efforts, HF remains frustratingly difficult to diagnose and prevent, and the (re-) admissions for HF after a HF diagnosis remain common [24]. There is considerable interest in using technologies to extend the clinical HF monitoring to the home, so as to enable more frequent assessment and treatment before the patients’ symptoms progress to the point of requiring hospitalization [24,25]. Hence, a reliable, noninvasive, easy to use, affordable, and automated HF detection device, that is acceptable by chronic HF patients, is urgently needed.

A possible solution may lie with a TBI vest, which was reported to offer some promising results [6,26]. However, the main drawback of the TBI vest that was reported was the textile electrodes, which required wetting prior to their use and frequently shifted when a subject moved, which compromised the accuracy of the impedance measurements. Thus, we examined whether our recently-developed carbon/PDMS electrodes could be used instead of the textile electrodes to overcome these limitations. Specifically, the main goal of this work was to determine the consistency of the TBI measurements that were provided by three types of carbon-black based electrodes, textile electrodes (Philips), and adhesive electrodes (Ag/AgCl). Furthermore, we have analyzed the amplitudes of the ECG signals that were obtained using each type of electrode. Although we didn’t run the AF detection algorithm because, for this study, all of our subjects were healthy, the amplitudes and quality of the obtained ECG signals were suitable for such an algorithm. The preliminary results of the study have previously been presented in a conference [27].

## 2. Materials and Methods

### 2.1. Transthoracic Bioimpedance (TBI)

The TBI was a painless and relatively simple measurement method that was used to determine abnormal changes in the amount of fluid in the body, as well as to estimate thebody composition. To measure TBI, typically four electrodes were used to compensate for the impedance of the electrodes and the electrode-tissue interface. They were placed in pairs on opposite sides of the lower lateral aspect of the thorax. Two electrodes on one side were used to inject an alternating current into the thorax, and the two opposite electrodes were used to measure the voltage drop across the thorax. Impedance was computed using Ohm’s law. The total impedance was comprised of two main components, namely, the baseline or constant component, which represented the impedance of the chest skeletal muscle, cardiac muscle, lung tissue, thoracic blood and plasma volume, chest wall fat, and air; and the dynamic component, which was caused by changes in impedance that were produced by the blood volume and velocity in the thoracic aorta. Only the constant component was dependent on the total thoracic tissue. Given that the thoracic fluid (intra- and extra-vascular) was the most conductive and variable component of the thoracic tissue, the baseline component was primarily modulated by the changes in fluid [28]. As fluid increased in the thorax, the impedance decreased (i.e., conductance increased), and vice versa. 

The described model had some limitations. The patients needed to be between 1.2 m and 2.1 m in height and between 30 kg and 155 kg in weight, in order to attain an accurate calculation of the stroke volume [29]. The presence of an intra-aortic balloon pump, aortic regurgitation, advanced sepsis, or extreme tachycardia might have invalidated the TBI measurements [29]. Originally, it was believed that the lung was the source of the signal [30]; however, later on, the aortic volume pulsation was thought to be the source of the signal [31,32]. More recent studies found that the aorta contributed to only about 1% of the total impedance measurement, the skeletal muscle represented the highest contribution (>50%), and the measure was highly sensitive to changes in impedance in the upper thorax [33,34]. Despite the limitations and the additional components that were captured by the TBI, it had clinical importance, as the intra-patient directional changes in the impedance were indicative of directional changes in the thoracic fluid [35].

**Figure 1:**
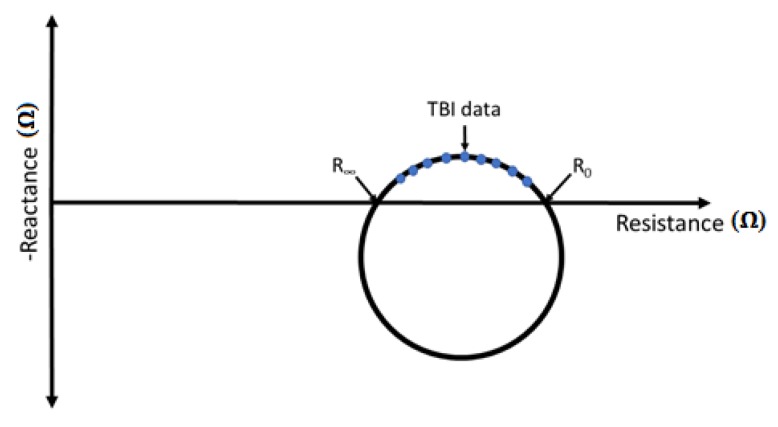
Illustrative example of Cole–Cole plot for Transthoracic Bioimpedance (TBI) measurements.

Figure 1 shows how the reactance and resistance that were measured with the TBI measurements changed with frequency. Impedance (resistance and reactance) was measured at different frequencies, and the result of each measurement was mapped into a resistance–reactance point on the graph. The measurement frequency increased counterclockwise in the graph, which meant that the lowest frequencies corresponded to the points farthest to the right.

**Figure 2:**
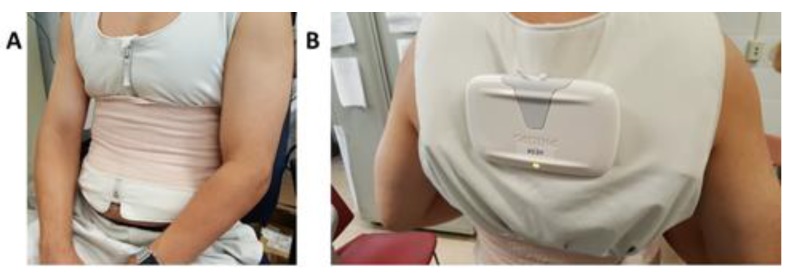
Subject wearing a fluid accumulation vest (FAV): (**A**) Front of the vest showing the compression strap and (**B**) measuring device connected via Bluetooth to a smartphone.

It was possible to derive an electric equivalent model for the biological tissue, with a resistor *R*_0_, which represented the extra-cellular fluid that was in parallel with a resistor *R_I_*, which represented the intra-cellular fluid, and a capacitor *Cm*, which represented the cell membranes. Using this model, it was possible to show mathematically that the points should lie on the arc of a circle, as shown in Figure 2. This was called the Cole–Cole plot [36,37,38]. At zero frequency (*f* = 0), the current could not pass the capacitance *Cm* (cell membrane), so it rans around the cells in the extra-cellular fluid. In this extreme case, the total impedance of the model was only *R*_0_. At the other extreme, at infinite frequency (*f* = ∞), the current would have passed through capacitance without reactance (cell membranes), and hence would have run through the cells in the intra-cellular fluid and in the extra-cellular fluid. In such a case, the impedance of the model was the parallel of *R*_0_ and *R_I_*. The total resistance was called *R_∞_*. *R_I_* could be calculated as follows: *R_I_* = (*R*_0_ × *R_∞_*)/(*R*_0_ − *R_∞_*).

*R*_0_ and *R_∞_* could be extrapolated from a set of measurements that were made at a predefined set of frequencies. For each frequency, the real (resistance) and imaginary (reactance) part of the electrical impedance was estimated. The extrapolation routine was based on a combination of the algebraic Taubin algorithm [39], which solved a circle fitting problem, and the Nelder–Mead algorithm [40], which was a heuristic search method. The Taubin algorithm was used to fit a circle onto the measured impedance data. From the circle data that were computed using the Taubin algorithm, the parameters of the Cole model were estimated using the Nelder–Mead algorithm. 

As for the clinical significance, *R*_0_ and *R_I_* were used to assess the intra- and extra-cellular fluids [41,42]. A low amount of extra-cellular fluid would have resulted in a high value of *R*_0_. Likewise, a low amount of intra-cellular fluid would result in a high value of *R_I_*. As the relative amount of fluids could be directly inferred from these two measures, *R*_0_ and *R_I_* were computed in this study from each TBI recording.

### 2.2. TBI and ECG Data Collection Protocol

**Figure 3:**
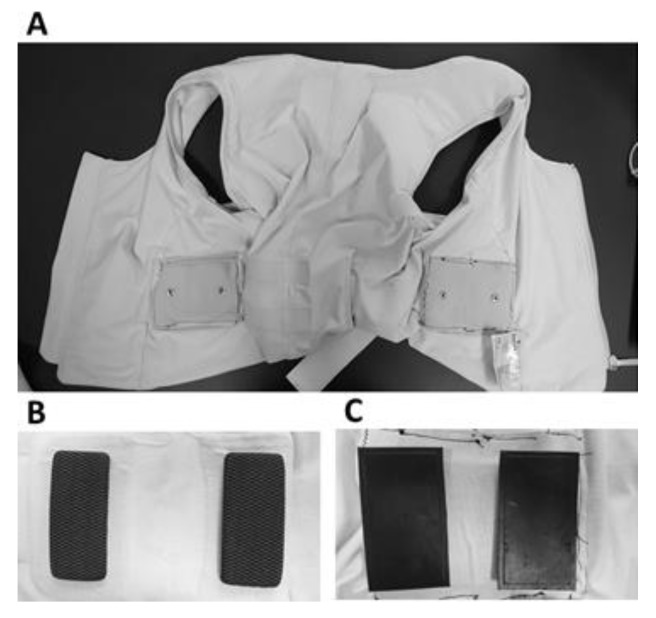
FAV and electrodes. (**A**) The FAV (Philips) modified to include snaps for electrode connections; (**B**) textile electrodes embedded in a Philips vest; and (**C**) carbon black polydimethylsiloxane (CB/PDMS) electrodes.

Twenty healthy volunteers (12 males and 8 females) of ages ranging from 18 to 54 years old (26.4 ± 9.2; mean ± SD), weight 67.6 ± 13.5 kg, and height 172.5 ± 9.7 cm, were enrolled in this study. The experiments were carried out in a quiet, dimly lit room (ambient temperature, 26–27 °C). At the start of the test, each subject was asked to scratch their skin on the left and right side of the abdomen with a plastic device, so as to remove any undesirable excess of dead skin cells. Before each TBI and ECG test, the subject’s skin at the location of the electrode placement was cleaned with 70% isopropyl alcohol. For this study, we used the fluid accumulation vest (FAV) (Philips, Amsterdam, the Netherlands) [6] (Figure 2 and Figure 3A), which provided TBI measurements at 16 frequencies in the range from 10 kHz to 999 kHz. The volunteers wore the FAV directly on their skin, so that its four electrodes were affixed to their abdominal region. The textile electrodes were wetted for this experiment. For each test, the volunteers were asked to sit still for five minutes for the recordings to be collected. Once the test was completed, the measuring device that was attached to the vest (Figure 2B) wirelessly transmitted the measurement data via a secure Bluetooth connection to a mobile phone (Samsung Galaxy Gio GT-S5660, Seoul, South Korea). The TBI and ECG data were recorded in an extractable SD memory card on the mobile phone and were subsequently transferred to a PC for processing and analysis. An elastic strap was used to assure a suitable pressure on the electrodes, while collecting the TBI and ECG data (Figure 2A).

A total of five types of the electrodes were tested for the collection of the TBI and ECG measurements. As a reference for comparison, the textile electrodes (Philips, Figure 3B) and commercially available Ag/AgCl (CLEARTRACE 1700-005, CONMED Corporation, New York, NY, USA) hydrogel adhesive electrodes were employed. Three types of CB/PDMS electrodes were tested, as follows:

1. Copper mesh CB/PDMS (CM/CB/PDMS);
2. Poly(3,4-ethylenedioxythiophene) polystyrene sulfonate (PEDOT) textile CB/PDMS (PT/CB/PDMS); and
3. PEDOT salt textile, CB/PDMS (PST/CB/PDMS).

The experimental procedure and setup lasted approximately two and a half hours per subject. The study protocol was approved by the Institutional Review Board of the University of Connecticut and all of the volunteers consented to be subjects in the experiment. All of the subjects gave written informed consent, in accordance with the Declaration of Helsinki. 

### 2.3. Fabrication of CB/PDMS Electrodes

The CB/PDMS electrodes were fabricated following the procedure that was described in our previous studies [10,11]. The following steps describe the fabrication procedure of the CB/PDMS with an embedded layer:

(A) The 3D printed Acrylonitrile Butadiene Styrene (ABS) cavity molds (Objet350 Connex, Stratasys, Eden Prairie, MN, USA) were filled with the conductive CB/PDMS composite and leveled so that no excess material remained.
(B) A highly conductive layer was affixed on the CB/PDMS mixture to allow a signal acquisition via the monitoring device. An insulated and waterproofed wire was soldered to the material of the highly conductive layer and was used as a connector to an ECG monitoring device. Three different conductive materials were tested on the CB/PDMS electrodes for the TBI and ECG measurements with the FAV, as follows: (1) copper mesh; (2) poly(3,4-ethylenedioxythiophene) polystyrene sulfonate (PEDOT) screen printed synthetic leather; and (3) PEDOT-salt screen printed synthetic leather.
(C) PDMS and curing agent mixture was then used to encapsulate the exposed surface with either an embedded copper mesh, PEDOT only leather, or PEDOT-salt leather.
(D) All of the components were degassed for 15 min in a vacuum chamber.
(E) The fasteners were soldered to the exposed end of the wire that was extended from the electrode.
(F) The completed electrode assembly was then placed in a curing oven at 75 °C for 3 h.
(G) After 3 h, the molds were removed from the curing oven and subsequently the electrodes were also removed from the cavity molds.

The resulting CB/PDMS electrodes were named, depending on the applied conductive layer, as described above. The appearance of the CM/CB/PDMS, PT/CB/PDMS, and PST/CB/PDMS electrodes was identical (Figure 3C), as the variant was encapsulated. We modified each FAV, as it was needed, from the embedded textile electrodes (Figure 3B) to one of the three aforementioned types of electrodes for TBI measurements (see Figure 3C). Note that the textile electrodes that are shown in Figure 3B were embedded into the vest. The carbon-based electrodes were placed onto the vest, using a snap connector to each electrode, shown in Figure 3A. Similarly, the Ag/AgCl electrodes were connected to the vest using snap connectors. 

### 2.4. TBI and ECG Measurements

**Figure 4:**
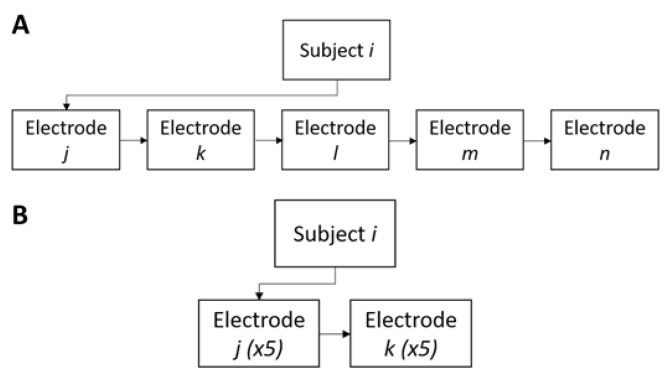
Flow charts of inter-subject (**A**) and intra-subject (**B**) analyses.

In this study, we tested the feasibility of carbon black/polydimethylsiloxane (CB/PDMS) electrodes as an alternative to the adhesive and textile sensors for measurement of TBI and ECG data. Specifically, we conducted two experiments to study the consistency and reliability of the bioimpedance measurement data that were acquired with three versions of carbon black electrodes, textile electrodes, and the gold standard silver-silver chloride (Ag/AgCl) hydrogel adhesive electrodes. Firstly, we assessed the repeatability of TBI and ECG measurements between the subjects, using adhesive, textile, and three types of CB/PDMS electrodes (inter-subject analysis). Subsequently, the two electrodes that exhibited the best performance were compared in terms of their consistency of TBI measurements when the measures were collected repeatedly for the same subject (intra-subject analysis). The flow charts of these two experiments are shown in Figure 4. The same *N* = 20 subjects underwent both of the tests. 

#### 2.4.1. Inter-Subject TBI and ECG Tests

Each subject was asked to wear the FAV in order to collect the TBI and ECG, using each of the types of electrodes sequentially and always in the same order, as follows: (1) Ag/AgCl electrodes; (2) textile electrodes; (3) CM/CB/PDMS; (4) PT/CB/PDMS; and (5) PST/CB/PDMS. One measurement was collected with each type of electrode. The placement of all of the types of the electrodes was identical for each subject and all of the types of electrodes, by marking their placement on the skin surface with a pen. All of the subjects were measured using the different electrodes, in the same order. To assess the inter-subject variability and degree of consistency of each index, the coefficient of variation (CV,$\frac{\sigma }{\bar{x}}$, unitless) was calculated. The differences between the resulting TBI measurements, compared with the textile electrodes, were determined using the Student’s t-test. The Bland–Altman plots were used to compare the differences in the measurements between the tested and textile electrodes (the five consecutive measurements that were obtained with the textile electrodes for the intra-subject consistency test were used as a reference). Only the results for the CM/CB/PDMS electrodes have been shown, as they provided the lowest CV values when compared with the two other types of electrodes.

To test the feasibility of recording the ECG waveforms and performing the AF detection from those, amplitudes of the R-peaks were compared between recordings made with different electrodes. The most popular approach for AF detection was based on the variability analysis of the heart beat intervals. For example, the disorganized atrial electrical activity that characterized the AF generated a random sequence of heart beat intervals with markedly increased beat-to-beat variability. This variability could be measured from the detected R peaks of the ECG recordings. Furthermore, we recently developed an accurate AF detection algorithm that searched for patterns of randomness in the AF [43,44,45]. 

#### 2.4.2. Intra-Subject Consistency Test

For this test, two types of electrodes were used to perform the repeated TBI measurements, always in the same order, namely: (1) five measurements with textile electrodes and (2) five measurements with CM/CB/PDMS electrodes. After each measurement, the subjects were asked to completely remove the FAV, the skin and electrodes were cleaned with alcohol, the skin was allowed to dry, and the FAV was placed again for the next measurement. For each subject, the measurements were taken at the same body location for both of the types of electrodes. This part of the study aimed to explore the consistency of the TBI measurements that were obtained using the electrodes under the test, for the same subject, in a short period of time, when the measurements should have exhibited low variations. The intra-subject CV and the intra-class correlation (ICC) were computed for *R*_0_ and *R_I_*, respectively. The ICC was computed as it was defined in the literature [46], for the *N* = 20 independent subjects, using the five independent measurements.

## 3. Results

### 3.1. Inter-Subject TBI and ECG Tests

**Figure 5:**
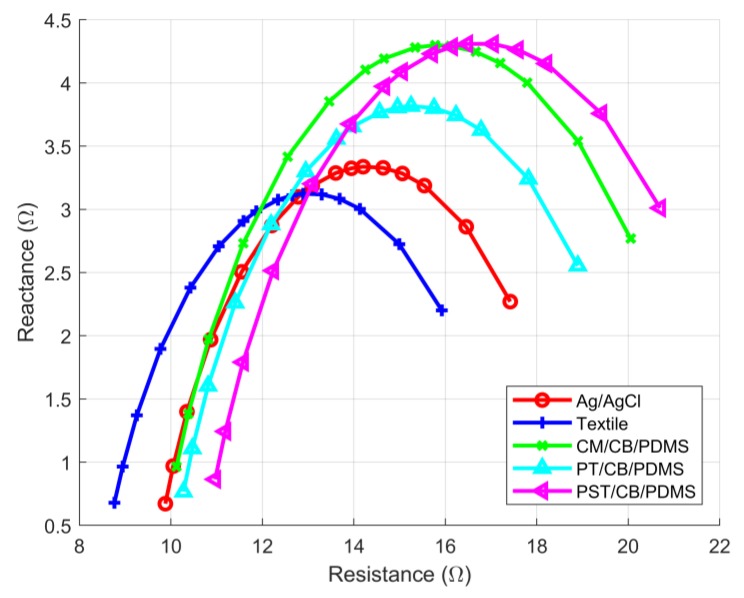
Cole–Cole plots obtained for the five types of electrodes, for a given subject.

Figure 5 shows the Cole–Cole plot for a given subject. The curves resulted from the polynomial regression of multiple measurements that were carried out at 16 pre-determined frequency values. Table 1 shows the resulting *R*_0_ and *R_I_* values that were computed for all of the electrode types from the *N* = 20 subjects. As shown, the results of the compared electrodes all significantly differred from the results that were obtained with the textile electrodes. The textile electrodes consistently provided the lowest value of *R*_0_ and *R_I_* for all of the subjects (Table 1). The Ag/AgCl electrodes provided a Cole–Cole plot of a similar amplitude (max-min of inverted reactance) compared to textile electrodes, but it always shifted towards the higher resistance (right-hand side). All three types of CB/PDMS-based electrodes showed higher differences in reactance and their achieved values for resistance were always higher when compared with the textile electrodes. 

For the measures of consistency, *R*_0_ exhibited a lower variation among the tested electrodes, when compared to *R_I_*. Table 2 contains the results of the coefficient of variance (CV) analyses. The lowest CV for *R*_0_ was achieved with textile electrodes, followed by Ag/AgCl, CM/CB/PDMS, and PST/CB/PDMS. The measures of *R_I_* exhibited higher variations, as the CVs were much higher for the textile, PT/CB/PDMS, and PST/CB/PDMS electrodes, while the Ag/AgCl and CM/CB/PDMS electrodes achieved lower CV values. The Ag/AgCl and CM/CB/PDMS electrodes exhibited the lowest variations when both *R*_0_ and *R_I_* were considered. The Bland–Altman results were in agreement with the CV analysis (Table 3). The mean value of the five consecutive measurements with textile electrodes were used as a reference. The CM/CB/PDMS electrodes achieved the highest *r*^2^ for bothof the measurements, the lowest coefficient of repeatability (CR) for *R*_0_, and the second lowest measurement for *R_I_*. The bias of the CM/CB/PDMS electrodes was higher compared with the textile electrodes, for both of the resistance indices. 

**Figure 6:**
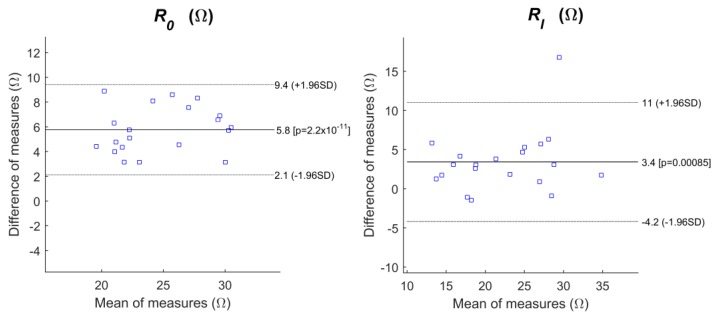
Bland—Altman plots for the copper mesh carbon black polydimethylsiloxane (CM/CB/PDMS) electrodes vs. textile electrodes. Left: *R*_0_; right: *R_I_*.

Figure 6 shows the Bland–Altman plots for the CM/CB/PDMS electrodes vs. textile electrodes, for *R*_0_ and *R_I_*, as the CM/CB/PDMS electrodes exhibited the results that were most comparable to the textile electrodes among the dry electrodes that were tested in this study. The *y*-axis represented the difference of measures, using different electrodes on the same subject and the *x*-axis represented the mean of the two measures. Note that the interval coefficient for *R*_0_ that was obtained with CM/CB/PDMS did not contain zero, which suggested that the values of *R*_0_ were not affected by the difference in the electrodes.

**Figure 7:**
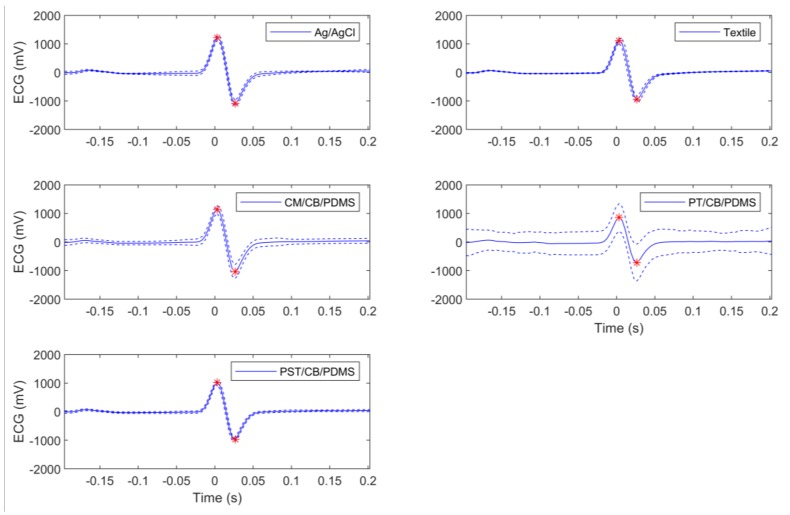
ECG templates for a given subject. Solid line is the resulting template. Dotted lines represent mean ± one standard deviation. Red marks represent maximum and minimum values of reference to compute peak-to-peak amplitude.

The general results for peak-to-peak amplitudes are in Table 1, while Figure 7 illustrates the results for a given subject. No significant differences were found between the tested electrodes. The highest mean amplitudes were achieved by the textile and Ag/AgCl electrodes, followed by the CM/CB/PDMS and PST/CB/PDMS electrodes. 

### 3.2. Intra-Subject Consistency Test

**Figure 8:**
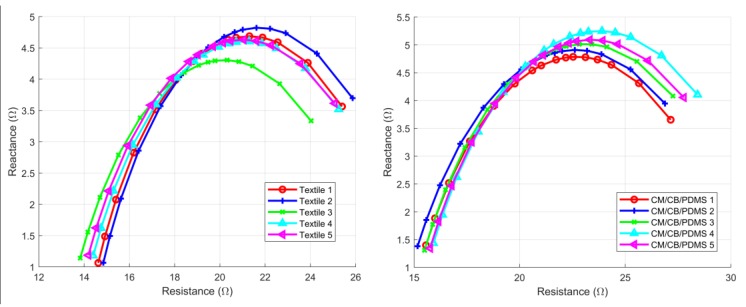
Intra-subject measurements for a given subject. Left: textile electrodes. Right: CM/CB/PDMS electrodes.

Table 4 includes the results for the intra-subject consistency analysis, while Figure 8 shows the results for a given subject. No statistically significant differences were found for the CV between the textile electrodes and the CM/CB/PDMS electrodes. The textile electrodes exhibiteda lower mean CV for *R*_0_, while the CM/CB/PDMS showed a lower mean CV for *R_I_*. ICC was high for both of the electrode types, and slightly higher for the textile electrodes.

## 4. Discussion

**Figure 9:**
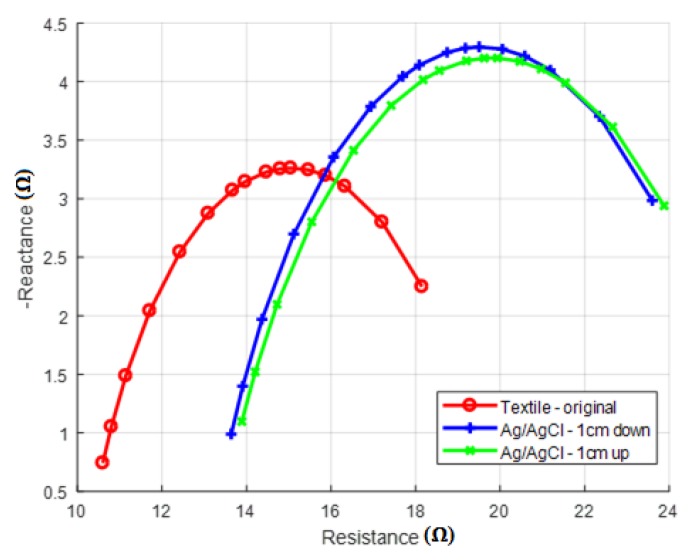
Results moving the Ag/AgCl electrodes.

In general, the textile electrodes that were embedded into the original vest detected the lowest *R*_0_ values. The relation between the textile and Ag/AgCl electrodes was that the textile showed a lower impedance at zero frequency, which could have been partially because of the smaller electrode-skin contact surface of the Ag/AgCl electrodes. The difference could also have been partly explained by the location and electrode material. To further explore what could have caused this difference, we performed TBI measurements with the Ag/AgCl electrodes at a given location, then moved them vertically 2 cm lower, so that we made clearly different points of measurement. Afterwards, we made a measurement with the textile electrodes at a location that was centered between the two locations where the Ag/AgCl electrodes were placed, so that the impact of the placement on the thorax of the Ag/AgCl electrodes and how that related to the measurement with the textile electrodes could have been observed. The obtained results are shown in Figure 9. As shown, the Cole–Cole plots for both of the placements of the Ag/AgCl electrodes were nearly identical. This led us to conclude that the location of the electrodes was not the cause of the lower values that were obtained with the textile electrodes, but the fabricated material itself.

With regard to the repeatability, we hypothesized that the textile electrodes showed better repeatability because they were sewn on the vest, while the CM/CB/PDMS electrodes had the problem of a possible rotation of the electrodes around the centrally located connector, as it was noticed in some of the instances of our measurements. This would introduce a variation in the tissue under the electrode that was to be measured, which would have been seen as variation in the measurement results. Sometimes we obtained equally good repeatability, as with the textile electrodes, which indicated that when the position of the electrodes was well controlled, the measurement results were equally good. To solve the positioning problem, in future studies, the electrodes should be sewn on the vest completely, or at least, should use two snap buttons to make them insensitive to rotation.

As shown in Table 3 and Figure 6, the CM/CB/PDMS electrodes achieved the best fitted polynomial regression to the textile electrodes (highest *r*^2^). As textile electrodes were the standard reference of stability, because of the fact that they were affixed to the measuring TBI vest, the best *r*^2^ meant that the CM/CB/PDMS electrodes provided highly stable measurements even when they were not affixed to the vest. The CM/CB/PDMS electrodes also exhibited a low CR, which was desirable because the CR was computed as 1.96 times the standard deviation of the differences between the CM/CB/PDMS and textile electrodes, and represented the agreement between both of the media. Although the bias between the CM/CB/PDMS and textile electrodes was the highest, the intended aim was not to produce identical TBI measurements to ones that were obtained with the textile electrodes, but the most consistent and reliable measurements of TBI, as these measurements were interpreted based on the trend, not the absolute value.

## 5. Conclusions

In general, we have shown that the carbon-black based electrodes are a suitable alternative to the textile electrodes for the TBI measurements using the FAV. Furthermore, they can collect ECG signals with an amplitude appropriate to deploy the AF detection. Embedding CM/CB/PDMS electrodes into the fabric will even further improve the consistency of the measurements that are already comparable to the ones that have been obtained with the textile electrodes.

## Figures and Tables

**Table 1:** Resulting *R*_0_ and *R_I_* and peak-to-peak electrocardiogram (ECG) amplitudes for inter-subject analysis.

Electrode Type	*R*_0_ (Ω)	*R_I_* (Ω)	Peak-To-Peak ECG Amplitude (V)
Ag/AgCl	24 ± 3.7 *	24 ± 5.2 *	1.53 ± 0.69
Textile	21 ± 3.1	19 ± 8.2	1.55 ± 0.63
CM/CB/PDMS	26 ± 4.5 *	24 ± 5.6 *	1.51 ± 0.74
PT/CB/PDMS	26 ± 6.6 *	25 ± 11 *	1.44 ± 0.82
PST/CB/PDMS	26 ± 5.4 *	27 ± 13 *	1.5 ± 0.69

Values are expressed as mean ± standard deviation. * Statistically-significant difference with respect to the textile (*p* <0.05). CM/CB/PDMS—copper mesh carbon black polydimethylsiloxane; PT/CB/PDMS—poly(3,4-ethylenedioxythiophene) polystyrene sulfonate (PEDOT) textile CB/PDMS; PST/CB/PDMS— PEDOT salt textile, CB/PDMS.

**Table 2:** Coefficient of variation (unitless) for the different electrodes.

Electrode Type	*R*_0_	*R_I_*
Ag/AgCl	0.15 (0.14 0.16)	0.22 (0.2 0.24)
Textile	0.15 (0.14 0.16)	0.42 (0.39 0.46)
CM/CB/PDMS	0.17 (0.16 0.18)	0.24 (0.22 0.26)
PT/CB/PDMS	0.25 (0.23 0.27)	0.42 (0.39 0.46)
PST/CB/PDMS	0.2 (0.19 0.22)	0.50 (0.46 0.55)

Confidence interval of the coefficient of variation (CV) is provided with a level of significance of 0.05.

**Table 3:** Bland–Altman analysis for inter-subject concordance. Textile electrodes were used as reference.

	*r* ^2^	Bias (CI) (Ω)	CR (Ω)	sd (Ω)
	***R*_0_**			
Ag/AgCl	0.6940	2.3 (−1.9 6.5)	4.2	2.1
CM/CB/PDMS	0.7937	5.8 (2.123 9.4)	3.6	1.9
PT/CB/PDMS	0.5310	4.5 (−4.6 13.6)	9.1	4.6
PST/CB/PDMS	0.6707	4.3 (−1.9 10.5)	6.2	3.1
	***R_I_***			
Ag/AgCl	0.7867	3.2 (−2.3 8.6)	5.4	2.8
CM/CB/PDMS	0.6915	3.4 (−4.2 11.0)	7.6	3.9
PT/CB/PDMS	0.1456	4.9 (−14.9 24.8)	19.8	10.1
PST/CB/PDMS	0.5865	6.2 (−12.8 25.2)	18.9	9.7

*r*^2^—coefficient of determination; CI— confidence interval provided with a level of significance of 0.05; CR—1.96 × sd, coefficient of repeatability; sd—standard deviation.

**Table 4:** Intra-subject consistency analysis results.

	CV (CI)		ICC (CI)	
Electrode	*R*_0_	*R_I_*	*R*_0_	*R_I_*
Textile	0.034 (0.029 0.04)	0.058 (0.048 0.067)	0.99 (0.98 1)	0.99 (0.98 0.99)
CM/CB/PDMS	0.041 (0.035 0.048)	0.079 (0.066 0.091)	0.98 (0.97 0.99)	0.96 (0.92 0.98)

CV—coefficient of variation; ICC—intra-class correlation coefficient; CI—confidence interval provided with a level of significance of 0.05.
